# China’s new initiative on basic research

**DOI:** 10.1093/nsr/nwag406

**Published:** 2026-07-09

**Authors:** Mu-ming Poo

While the global community is marveling at China’s impressive advances in many technology sectors, the Chinese government is planning a renewed focus on further strengthening basic research. On 30 April, President Xi Jinping gave a speech at a symposium in Shanghai on strengthening basic research. He urged greater efforts and more concrete measures to further strengthen basic research in order to enhance China’s capacity for original innovation and to solidify the foundation for the country’s strength in science and technology (S&T).

Several key points in President Xi’s speech are particularly noteworthy. He acknowledged that a new round of S&T revolution and industrial transformation is accelerating, and that global S&T competition is increasingly centered on frontier areas of basic research, where original and disruptive innovation is becoming increasingly prominent. He calls for strengthening overall planning and top-level design to optimize the systematic layout of basic research, by further clarifying the main targets and key areas of basic research. It is generally recognized that basic research is the fountain for new technologies. Yet there are persisting debates on whether disruptive innovation could be planned and whether the main targets and key areas of basic research could be designed. A wide-spread opinion in many science areas is that ‘free exploration’ driven by individual scientist’s interest-without top-down directives is the norm of doing basic science, as scientific breakthroughs were often made in a haphazard and unexpected manner.

This view of basic research as unplanned free exploration driven only by individual scientist’s interest is not in line with governmental leaders’ view on the role of basic research. As articulated clearly by President Xi on 11 September 2020 in a forum of scientists, China’s S&T development is oriented toward Four Directions (四个面向)—the world’s S&T frontiers, the main economic fields, the country’s major needs and people’s life and health—toward which basic research for original discoveries and inventions should contribute. Thus, basic research must have clear overall goals, even though the paths to achieve them could be varied and subjected to free exploration.

In an affluent society with abundant resources, basic research scientists could be free to pursue their personal interests and satisfy their own curiosity without considering whether the outcome meets the society’s needs. Biologists enjoyed such freedom in the USA during the last quarter of 20th century. Each year, National Institutes of Health awarded tens of thousands of research grants, without demanding research outcome. Several Nobel laureates have emerged among the awardees, drug targets were identified and a few expensive therapies have been developed. Yet, the great majority of the grants yielded no real return to the society and provided little contribution to the health of the majority of people. It is clear that neither China nor any other developing country should and could afford to support such a research enterprise.

Basic research in scientific versus technological fields could differ in the degree to which the goals and research plans could be clearly defined. A case in point is the recent successful deployment of the 2-MW thermal thorium-based molten salt reactor, the first operational fourth-generation nuclear reactor, in Gansu Province. To achieve successful conversion of thorium-232 into fissile uranium-233 and 100% operational capacity, many clearly defined basic scientific problems were solved by focused research. In the celebrated breakthrough in basic biology—the discovery of induced pluripotent stem cells—the goal and research plan were clear in the discoverer Yamanaka’s mind as he explored various factors that may achieve the de-differentiation of somatic cells. Similarly, recent discoveries of rejuvenation factors that reverse the aging of somatic cells also depended on goal-directed exploration in basic research laboratories. It is thus an overstatement that important S&T breakthroughs all depend on free exploration without goals and planning; however, they may seem vague and ill-defined initially.

President Xi’s emphasis on basic research should be viewed within the context of the integration of academia and industry. He called for strengthening of coordinated development between applied and basic disciplines in order to promote the innovation chain that spans basic research, application development and commercialization. He proposed a systematic approach to developing major S&T infrastructure, advancement of intelligent research platforms and improvement of a categorized evaluation system tailored to the characteristics of basic research; many of these have emerged as critical issues in China’s S&T establishment. Scientific talents are the driving force for basic research. President Xi advocated the spirit of scientists and the need for science dissimilation that could stimulate the imagination and curiosity of young people, so that pursuing basic research becomes their lifelong aspiration. He also stressed the need to strengthen support for basic research and called for gradually increasing the share of funds for basic research and establishing a mechanism of diversifying research resources.

Finally, basic research in any country is likely to benefit the global community as a whole, because the findings are published in international journals and accessible to all researchers. Notably, President Xi called for actively integrating Chinese basic research into the global innovation network, deepening international exchanges and cooperation in tackling major issues such as climate change, energy and environment, as well as life and health, and actively participating in global S&T governance. This is particularly noteworthy in view of the current geopolitical pressure for decoupling S&T development in Western countries from China and the recent emphasis on self-reliance in the Chinese research community. In the spirit of constructive strategic stability highlighted in the recent summit meeting between the US and Chinese presidents, we envision that cooperation in basic research could indeed constitute an important element in future international relationships.

